# Production of an anti-dermatophyte monoclonal antibody and its application: immunochromatographic detection of dermatophytes

**DOI:** 10.1093/mmy/myw037

**Published:** 2016-06-01

**Authors:** Sakon Noriki, Hisaya Ishida

**Affiliations:** 1 Department of Pathology, University of Fukui, Fukui, Japan; 2 Ishida Dermatology Clinic, Fukui, Japan

**Keywords:** trichophyton, monoclonal antibody, immunochromatography, diagnosis, dermatophytosis

## Abstract

Tinea refers to superficial infection with one of three fungal genera—*Microsporum, Epidermophyton, or Trichophyton*—that are collectively known as dermatophytes. These infections are among the most common diseases worldwide and cause chronic morbidity. They are usually diagnosed by direct microscopy and fungal culture, which are burdensome to perform in the clinical setting. To supplement conventional methods, we developed a new method that employs an immunochromatography test for detection of dermatophyte infections. First, anti-*Trichophyton* monoclonal antibodies (mAb) were produced in mice using a *Trichophyton* allergen solution as an immunogen. The mAb specificity was assessed by immunostaining alcohol fixed slide cultures and formalin fixed paraffin-embedded microbial samples. Both alcohol- and formalin-fixed samples of all seven species of *Trichophyton* tested displayed positive immunostaining. Immunochromatography test strips were created using the anti-*Trichophyton* mAb. The efficiency of the test strip was assessed in patients diagnosed with tinea unguium and in healthy volunteers. Of the 20 patient nails tested, 19 tested positive and one tested negative, whereas of the 17 volunteer nails, only one tested positive. However, KOH microscopic examination of the volunteer nail that tested positive revealed the existence of *Trichophyton* hyphae. Although the number of nails assayed was small, since the assay had a sensitivity of 95.0% (19/20) and a specificity of 94.1% (16/17), the obtained results were considered to be promising. Thus, while further investigation with a greater number of samples is necessary, this method could potentially be employed as a new diagnostic tool for *Trichophyton* in the future.

## Introduction

Tinea is caused by skin infection with dermatophytes and is a worldwide disease.^1^ It is estimated that 40% of the population is infected in Europe,^2^ and 20% of the population is infected in Japan.^3^ Medical treatments for tinea have progressed so well that tinea can be completely cured by a proper diagnosis and the appropriate treatment. Although many diagnostic methods have been proposed for diagnosis of tinea, the KOH direct microscopy method and the microbial culture method are the standard methods used in the clinic.^4^ However, the culture method is time-consuming, and there is the likelihood of obtaining negative results. Therefore, in reality, positive diagnosis of tinea depends on the KOH microscopy method. This method requires skill in identification of fungal hyphae without mistaking a fibre for a hypha. Since these tests are sometimes too burdensome to be performed in the clinical setting, we developed a new method of diagnosis to supplement the conventional methods. This method employs an immunochromatography test for detection of dermatophyte infections.

Immunochromatography tests that use an antibody for diagnosis of an infectious disease have been widely used as quick diagnostic methods. Kits that use an immunochromatography method for diagnosis of infection include kits for diagnosis of infections such as *Streptococcus*,^5^ *Haemophilus*,^6^ Adenovirus,^7^ and Norovirus.^8^ In particular, an influenza infection kit^9^ is used worldwide.

We therefore tried to design an immunochromatography test that uses an antibody for the diagnosis of tinea. There have been some previous reports regarding the production of a monoclonal antibody against dermatophytes.^10,11,12^ However, although there are a lot of commercially available antibodies against bacteria, there is no commercially available antibody against *Trichophyton*. We therefore produced a monoclonal antibody against *Trichophyton* (dermatophyte) and examined the specificity of this monoclonal antibody. Furthermore, we made a test strip with the newly produced monoclonal antibody against *Trichophyton* using the immunochromatography method and validated the usefulness of this test strip for the detection of dermatophytosis.

## Materials and methods

### Production of monoclonal antibodies (mAbs)

The monoclonal antibody against *Trichophyton* was produced by reference to a textbook.^13^ Since the details of this procedure have been described in a previous patent publication,^14^ the details are only briefly indicated here.

#### Immunisation

*Trichophyton rubrum* allergen (20,000 Protein Nitrogen Unit (PNU)/ml; Greer Laboratories, Inc., Lenoir, NC) was used as the immunogen for monoclonal antibody production.

#### Antibody evaluation ELISA plate

A solution of the *Trichophyton rubrum* allergen was diluted with phosphate buffered saline (PBS) to a titre of 200 PNU/ml, and 50 μl was placed in each well of a 96 well ELISA plate (Corning Inc., Corning, NY). The plates were incubated at room temperature for 1 hour for immobilisation of the allergen on the plate. After removal of the liquid in the well, Block Ace (Snow Brand Milk Products, Tokyo, Japan), which was diluted 4 times with distilled water, was dispensed in a final volume of 300 μl into each well and incubated for 1 hour at room temperature in order to eliminate non-specific binding. The liquid in each well was then removed, and the wells were washed 3 times with PBS containing 0.05% (W/V) Tween20 (0.05% Tween20-PBS). Ab-containing solutions were then dispensed into the sample wells at a final volume of 50 μl per well and were incubated at room temperature for 1 hour. Following removal of the liquid from each well and three washes with 0.05% Tween20-PBS, horseradish peroxidase (HRP) labeled rabbit anti-mouse IgG antibody (DAKO, Tokyo, Japan), which was diluted 1:2,000 with 10% (vol/vol) Block Ace, was placed in each well at a final volume of 50 μl and incubated at room temperature for 1 hour. After removal of the liquid from the wells and three washes with 0.05% Tween20-PBS, a chromogen solution containing 3, 3′ -5 5″-tetramethyl benzidine (TMB) (DAKO) was dispensed in a volume of 50 μl into each well, and the colour was allowed to develop in a darkroom. The enzyme reaction was stopped by adding 50 μl of 2 M sulphuric acid to each well after 10 minutes. Colour formation was measured at the main wavelength of 450 nm, and absorbance of the solution in the wells of the plates was also measured at the sub-wavelength of 650 nm, using micro-plate readers. A well that did not contain immobilised antigen was similarly measured as a control.

#### Hybridoma preparation

For preparation of hybridomas, 100 μl of *Trichophyton rubrum* allergen that was diluted 1:2 with PBS was injected intraperitoneally into mice on day 0 and on days 41, 55, 56, and 57. Cells were extracted from the spleen on the 58th day and were fused with BALB/c myeloma cells (Sp2/0-Ag14) using 50% polyethylene-glycol 1500 liquid (Roche Diagnostics, Risch-Rotkreuz, Switzerland). Selective culture with hypoxanthine, aminopterin, and thymidine (HAT) was then performed for nine days at 37°C under 5% carbon dioxide. The procedures involving mice conformed to the guidelines for the care and use of laboratory animals of the Institute of Laboratory Animal Research, Commission on Life Sciences, National Research Council.^15^

Antibody titres of the hybridoma culture supernatants were determined using the antibody evaluation ELISA plate described above. The supernatant from the well containing hybridoma 0014 showed reactivity to both the *Trichophyto*n *rubrum* allergen and the *Trichophyton mentagrophytes* allergen (Greer Laboratories, Inc.).

#### Monoclonal antibody purification

The hybridoma 0014 was cultured in 200 ml hybridoma culture-medium. The obtained supernatant was applied to a protein A Sepharose fast-flow column (GE Healthcare, Little Chalfont, United Kingdom) that was first equilibrated with 50 mM boric acid buffer containing 3 M NaCl. Subsequently, bound antibody was eluted with 0.1 M citrate buffer pH 3, and 2 ml of eluate containing the antibody was obtained. Protein content was calculated by measurement of the absorption at OD 280 nm.

#### Nature of mAb

*Subtyping of antibody*. The subtyping reagent, Iso strip (Roche Diagnostics), was used for subtyping analysis of the antibody.

*Periodate and protease treatment of the antigen recognised by the mAb 0014.* The *T. rubrum* allergen was fixed on ELISA plates by physical adsorption. For periodic acid treatment of the allergen, a solution of 50 mM sodium periodate was added to the wells. For protease treatment of the allergen, 0.1 mg /ml of a pronase solution was added to the wells. After washing and blocking of the wells following treatment, the supernatant containing antibody that was diluted with PBS was reacted with the antigen in the well (the primary reaction). Colour development and ELISA measurements were performed as described above.

*Biotinylation of the purified antibodies.* The purified antibodies were biotinylated with Biotin-XX-sulfosuccinimidyl ester (Dojindo, Kumamoto, Japan). Biotin-labelled 0014 antibodies were exacted by filtration through an NAP-5 column (GE Healthcare). Details of this procedure have been described in a previous patent.^14^

### Detection of microorganisms using a sandwich ELISA method

#### Preparation of the microorganism samples

The fungi, *Trichophyton rubrum* (IFO 9185, IFO 32409), *Trichophyton mentagrophytes* (IFO 6202, IFO 32410), *Microsporum canis* (IFO 32463), *Epidermophyton floccosum* (IFO 32461), and *Candida albicans* (ATCC 90028), which were provided by the Osaka foundation, were cultivated using a Sabouraud dextrose agar slant (Becton Dickinson Co. Ltd., Franklin Lakes, NJ). The bacteria, *Escherichia coli* (IFO 13500), *Streptococcus faecalis* (IFO 12968), and *Bacillus subtilis* (IFO 3026), which were also provided by the Osaka foundation, were cultivated on Lysogeny Broth (LB) agar medium dishes. Obtained colonies were each picked with a sterile 10 μl disposable loop and suspended in 5 ml of Block Ace solution that had been diluted 1:10 with distilled water. The microbial containing solutions were then further diluted 1:5, 1:25, 1:125, and 1:625 and these dilutions were used as the sample. The same dilutions of the *T. rubrum* allergen (100 PNU/ml) were used as positive control samples.

#### Measurement of the samples using a sandwich ELISA method

The purified 0014 antibody was diluted with 50 mM carbonic buffer to a concentration of 10 μg/ml and placed in the wells of a 96 well ELISA plate (Corning, Inc.). After incubation at room temperature, the wells were washed three times. Microbial solutions or positive control solutions were put in the wells and incubated. Following incubation, the samples were removed and the wells were thoroughly washed. The biotinylated 0014 antibody solution that was diluted to a concentration of 1 μg/ml with Block Ace solution was then added to each well, using 50 μl per well, and reacted at room temperature. Colour development and ELISA measurements were performed as described above.

#### Examination of the specificity of the monoclonal antibody 0014 using immunostaining

For analysis of the microbial specificity of the monoclonal antibody 0014, *Trichophyton rubrum* (IFO 9185, IFO 32409), *Trichophyton mentagrophytes* (IFO 6202, IFO 32410), *Microsporum canis* (IFO 32463), *Epidermophyton floccosum* (IFO 32461) *Trichophyton mentagrophytes* (NBRC5466), *Trichophyton violaceum* (KMU 4127), *Trichophyton tonsurans* (KMU 4462), and *Microsporum gypseum* (RV 15250) were used as dermatophytes. The latter four micro-organisms were kindly provided by Professor Mochizuki of Kanazawa Medical School. *Aspergillus (A) fumigatus* (KMU4483), *A. niger* (ATCC6274), *A. tamarii* (NBRC), *A. oryzae* (NBRC4075), *Sporothrix schenckii* (KMU975), *Fonsecaea pedrosoi* (KMU3846), *Penicillium surantiogriseum* (NBRC5847), *P. digitatum* (NBRC7006), *P. funiculosum* (ATCC9896), *Fusarium oxysporum* (KMU4521), *and Candida albicans* (NBRC0917) were used as non-dermatophytes.

These micro-organisms were inoculated and passaged on Sabouraud agar (Becton Dickinson Co. Ltd.) at room temperature. A sliding culture was performed to obtain material for the experiments as follows. The Sabouraud agar plate was cut into an 8 mm square block. A block of this culture agar was placed on a sterilised slide, and each microorganism was inoculated into the four cornersx of this piece of culture agar. The inoculated agar was then covered with a sterile cover glass and incubated in a humidification box for one week at room temperature. The cover glass from the slide culture was then placed into 99.5% ethanol for fixation and subsequent immunostaining. Additionally, the agar was placed into a 30% formalin solution for fixation, and paraffin embedding of the agar was then carried out prior to immunostaining.

For immunostaining of formalin fixed sections, a 4 μm-thick section of agar with microorganism that had been formalin fixed and paraffin embedded was deparaffinised with xylene, which was then replaced with ethanol. After washing with water, the intrinsic peroxidase activity was blocked with a 0.03% H2O2 solution dissolved in absolute methanol at room temperature for 15 minutes and the section was then rinsed with phosphate-buffered saline (PBS). The section in PBS was heated in a microwave oven at 750 W for 15 min in order to perform heat-mediated antigen retrieval. For immunostaining of ethanol fixed sections, the ethanol fixed cover glass from the slide culture was pretreated with the intrinsic peroxidase activity blocking solution as above.

Both formalin fixed sections and ethanol fixed sections were then reacted at 4°C overnight with the purified antibody 0014, which was diluted to a concentration of 4 μg/ml with PBS. Detection of antibody reactivity was performed using a commercially available kit (Dako EnVision Plus-horse radish peroxidase (HRP) (DAKO) as follows. The sections were reacted with a goat-anti-mouse EnVision-HRP–enzyme conjugate for 30 min at room temperature, followed by rinsing with PBS. The peroxidase colour reaction was visualised by incubating the sections with the chromogen 0.02% 3-3-diaminobenzidine tetrahydrochloride (DAB; Sigma Chemical Co., St Louis, MO, USA) at room temperature for 10 minutes. The sections were then washed with water. After the immunostaining procedures were completed, the sections were lightly counterstained with Haematoxylin. As a negative control, the exact same procedure was performed without the primary antibody 0014.

### Production of an immunochromatographic test strip

#### Preparation of a detection pad with antibodies bound to a solid phase membrane

To produce a detection pad (DP), a nitroglycerine cellulose sheet (HiFlow Plus) (EMD Millipore, Billerica, MA) was cut to a size measuring 5 mm × 20 mm, and the 0014 antibody solution was linearly applied to the sheet at 10 mm from the lower end with BioJet Q3000 (Biodot, Irvine, CA) and dried at room temperature.

#### Preparation of a colloidal gold particle-labelled antibody and a reagent pad

The 0014 monoclonal antibody was mixed with gold colloid particle liquid (PL-Latex, 10%, particles of 40 nm in diameter; Agilent Technologies, INC., Santa Clara, CA, United States) to obtain gold colloid particle-labelled antibody as previously described.^16,17^

A reagent pad (RP) was made by immersing a glass fiber non-woven fabric (GFCP001050, 5 mm × 10 mm) (EMD Millipore) in 25 μl of the above-mentioned colloidal gold particle-labeled antibody followed by drying by ventilation.

#### Preparation of the immunochromatography test strips

The immunochromatography test strips were assembled as follows. The reagent pad containing the colloidal gold-labeled antibody was placed on the detection pad up to 2.5 mm from its lower end. A sample absorption pad (SP) was then piled up from the bottom edge of the reagent pad to a distance of 2.5 mm on the reagent pad. In addition, an absorbent pad (AP) (3MM Chr, Whatman) was placed on the detection pad up to 2 mm from its upper end, and a transparent tape was finally attached to the top and fixed thereon to give an immunochromatography test strip.

### Assay of clinical samples using the immunochromatography test strips

#### Target

Twenty pieces of nail, one from each of 20 patients with tinea unguium who were diagnosed using KOH microscopy methods by dermatologists at Fukui Medical School Hospital, were used as the clinical samples. For the control samples, 17 pieces of nail, one from each of 17 healthy volunteers were used. All patients provided informed consent for participation in the study.

#### Method

A piece of nail was put into an Eppendorf tube, and 300 μl of sterile distilled water was added. After heating in boiling water for 10 minutes followed by natural cooling, 120 μl of the sample solution was added to the strip test, and judgment of positive or negative immunoreactivity was carried out after 5 minutes by visual inspection.

#### Data analysis

The efficiency of the immunochromatography test strip was analysed utilising a 2 × 2 contingency table test for Fisher's exact probability test. Parameters such as sensitivity, specificity, positive predictive value (PPV), and negative predictive value (NPV) were calculated. These analyses were performed using Excel statistics software (SSRI Inc., Tokyo, Japan).

## Results

### Monoclonal antibody production and selection

The change in the antibody value of immune sera that were periodically collected from blood obtained from the tail veins of allergen-injected mice was measured using ELISA (data not shown). On the 53rd day after initiation of allergen injections, a rise in the anti-*T. rubrum* antibody titer to 1:120000 was observed. Among the cultivated hybridoma wells of hybridomas produced by selective culture, one clone was chosen from 5 wells. The antibody produced by this clone reacted with both *T. rubrum* and *T. mentagrophytes.* This antibody-producing clone was named 0014.

### The subtype of the monoclonal antibody 0014

**T**he subtype of the antibody 0014 was determined as IgG1 kappa (data not shown).

### Sugar/protein characteristics of the antigen reactivity with the monoclonal antibody 0014

The effect of sugar and protein degradation of the allergen by treatment with sodium periodate and pronase, respectively, on the reactivity of the antibody 0014 with the fixed allergen, was tested using an ELISA. There was no significant difference in the reactivity of the antibody with pronase-treated and non-treated allergen. However, the reactivity of monoclonal antibody 0014 with the allergen treated with sodium periodate was decreased compared to that with the non-treated allergen (Figure S1).

### The reaction of monoclonal antibody 0014 with various fungal and bacterial suspensions in a sandwich ELISA assay

In a sandwich ELISA assay, three species of bacteria (*Escherichia coli, Streptococcus faecalis, Bacillus subtilis*) and the fungus *Candida albicans* reacted with the antibody 0014 in a similar manner to the culture media, which was the negative control. On the other hand, *Trichophyton rubrum, Trichophyton mentagrophytes, Microsporum canis*, and *Epidermophyton floccosum* showed the same degree of reaction with this antibody as the *T. rubrum* allergen solution that was used as a positive control (Table S1).

### The specificity of the monoclonal antibody 0014 as assessed using immunohistological examination

**Figure 1.**
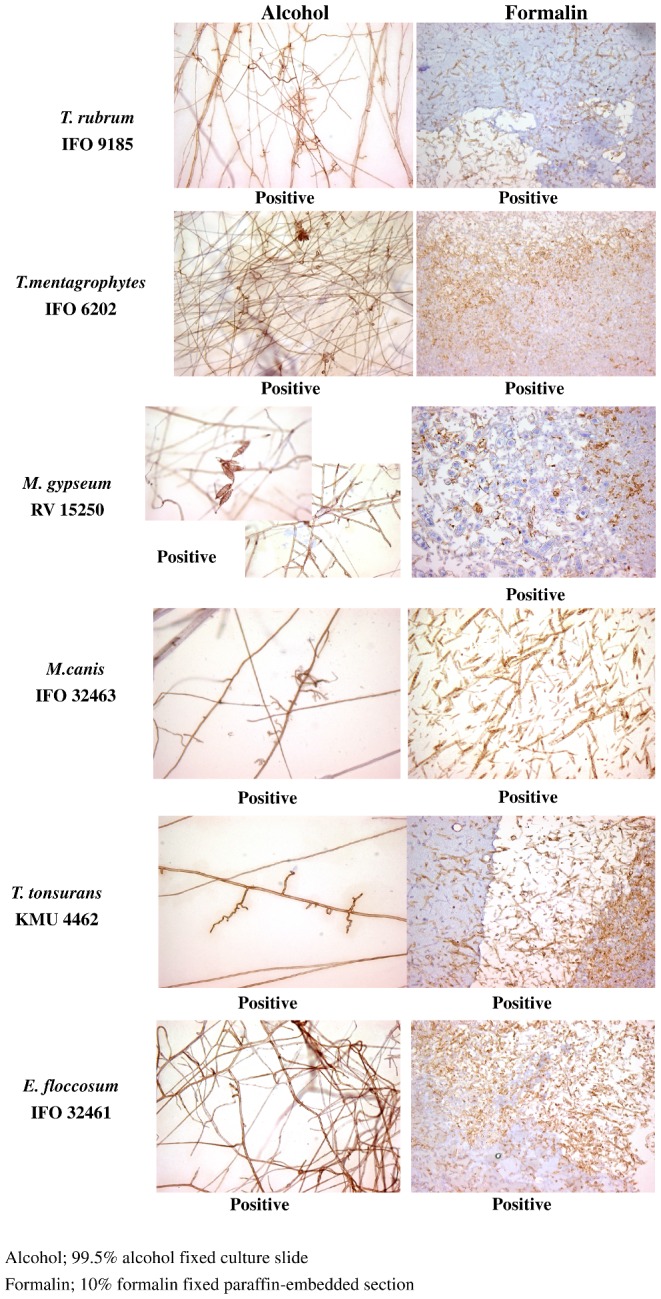
Results of the immunohistological staining of various fungi of the Trichophyton group with monoclonal antibody 0014. Reactivity with the antibody is summarised in Table 1.

**Figure 2.**
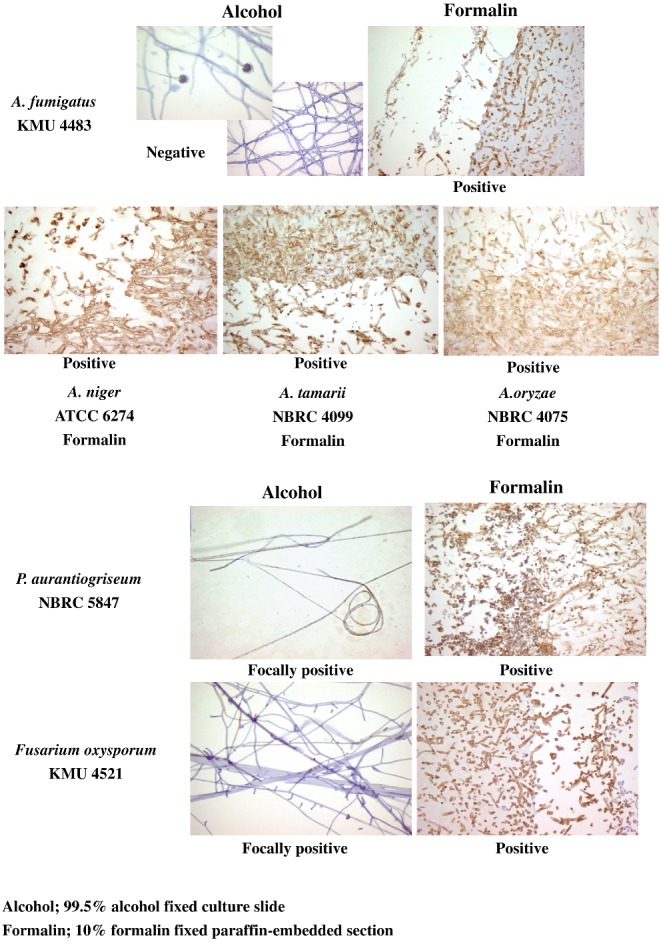
Results of immunohistochemical staining of various fungi of the non-Trichophyton group with monoclonal antibody 0014. Reactivity with the antibody is summarised in Table 1.

The results of immunohistological examination of the reactivity of monoclonal antibody 0014 with histological specimens of both alcohol fixed culture slides and formalin fixed paraffin embedded sections of various fungi of the *Trichophyton* group are shown in Figure 1. Both alcohol fixed culture slides and formalin fixed paraffin embedded sections of all seven species of *Trichophyton* showed positive reactivity with the antibody. In the slide culture specimens, both conidia and hyphae were stained. The results of similar assay of the reactivity of the members of the non-*Trichophyton* group with antibody 0014 are shown in Figure 2. *Penicillium aurantiogriseum* and *Fusarium oxysporum* of the non-*Trichophyton* group displayed focally positive reactivity in the alcohol fixed culture slide, but positive reactivity in the formalin fixed paraffin embedded section. *A. fumigatus, A. niger, A. tamarii*, and *A. oryzae* of the *Aspergillus* genus displayed positive reactivity only in the formalin fixed paraffin embedded section. Both *P. digitatum* and *P. funiculosum* had negative reactivity. *Sporothrix schenckii, Fonsecaea pedrosoi*, and *Candida albicans* were negative. Control sections treated without the primary antibody 0014 were also negative. These results are summarised in Table 1.

**Table 1.** Summary of the reactivity of various fungi with the 0014 antibody in immunostaining.

**Tinea Group**	**Alcohol**	**Formalin**
*Trichophyton rubrum*	Positive	Positive
*Trichophyton mentagrophytes*	Positive	Positive
*Trichophyton violaceum*	Positive	Positive
*Trichophyton tonsurans*	Positive	Positive
*Microsporum gypseum*	Positive	Positive
*Microsporum canis*	Positive	Positive
*Epidermophyton floccosum*	Positive	Positive
**Non-Tinea Group**	**Alcohol**	**Formalin**
*Aspergillus fumigatus*	Negative	Positive
*Aspergillus niger*	Not tested	Positive
*Aspergillus tamarii*	Not tested	Positive
*Aspergillus oryzae*	Not tested	Positive
*Sporothrix schenckii*	Negative	Negative
*Fonsecaea pedrosoi*	Negative	Negative
*Penicillium aurantiogriseum*	Focally positive	Positive
*Penicillium digitatum*	Negative	Negative
*Penicillium funiculosum*	Not tested	Negative
*Fusarium oxysporum*	Focally positive	Positive
*Candida albicans*	Negative	Negative

### Examination of antibody 0014 reactivity with clinical samples using immunochromatography test strips

**Figure 3.**
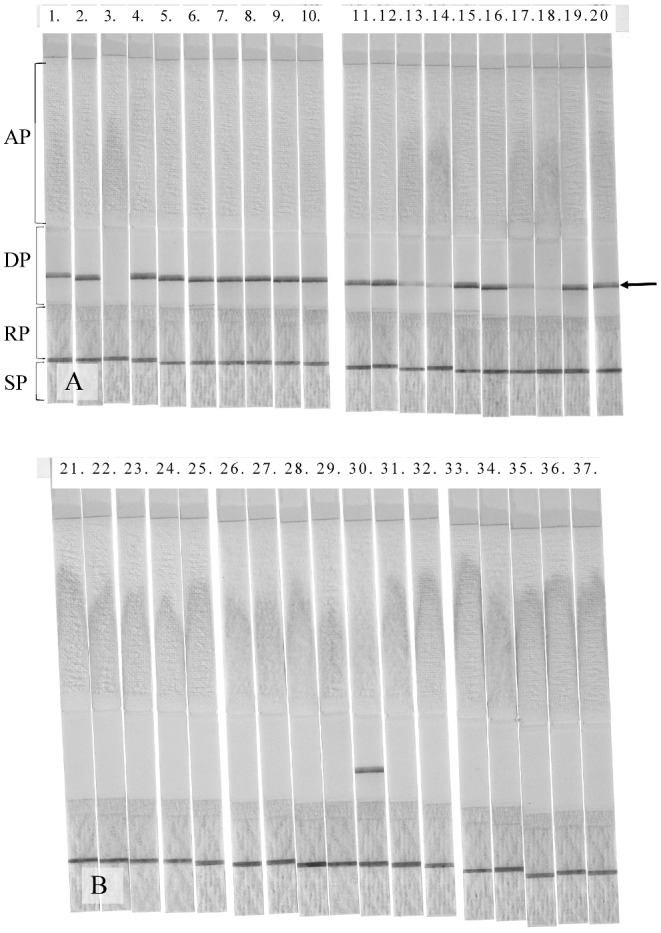
Analysis of patient and healthy nails using the immunochromatographic test strip. Results of immunochromatographic test strip analysis of the nails of (A) patients with tinea unguium and (B) healthy volunteers. The numbers at the top indicate the case number. The test strip is composed of a sample pad (SP), a reagent pad (RP), a detection pad (DP), and an absorbent pad (AP). The RP contains the anti-dermatophyte antibody conjugated with colloidal gold particles. The same antibody is immobilised in a transverse line on the DP. A positive signal is shown as a line on the DP (indicated by an arrow).

The results of immunochromatography test strip analysis of the reactivity of antibody 0014 with nail specimens from 20 patients who were diagnosed as tinea unguium are shown in Figure 3A. Nineteen cases displayed positive reactivity, and only one of the 20 specimens (case no. 3) was negative in terms of antibody reactivity. The results of immunochromatography test strip analysis of the reactivity of antibody 0014 with the nails of healthy volunteers are shown in Figure 3B. Of the 17 healthy subject nails assayed, only one case (case no. 30) displayed positive antibody reactivity.

Statistical analysis indicated that the two-tailed *P* value of Fisher's exact test was less than 0.0001. Furthermore, the sensitivity of the assay was .95 (95% CI 0.84–0.99), the specificity was 0.94 (95% CI 0.80–0.98), PPV was 0.95 (95% CI 0.84–0.99), and NPV was 0.04 (95% CI 0.80–0.98). Thus, it was considered that extremely high statistical significance was obtained for the association between the patient group or the healthy group and antibody 0014 positive or negative reactivity, respectively, in the immunochromatography test strip assay.

## Discussion

We produced a novel monoclonal antibody using the *T. rubrum* allergen as an immunogen. Both a sandwich ELISA method and an immunohistological method demonstrated that this monoclonal antibody reacted with all seven species of the *Trichophyton* group. Although this monoclonal antibody partially reacted with the non-*Trichophyton* group, reactivity was negative for *Candida*, which may exist as part of the normal flora of the skin. Therefore, this antibody can be called an anti-dermatophyte monoclonal antibody. The antigen which this antibody recognises is thought to be a soluble antigen from the fungus body of *Trichophyton*. Although an understanding of the nature of this antigen would be interesting, we were unable to identify it in this study. However, the antigen appears to be a carbohydrate moiety based on the fact that its reactivity with the antibody was not decreased by protease or heat treatment but was decreased by periodic acid treatment. Latgé et al. made rat monoclonal antibodies against exocellular carbohydrate antigens of Aspergillus and dermatophytes.^18^ They showed that the β(1-5)-galactofuran side chains of galactomannan are the epitope of their antibodies. Therefore, the epitope of our antibody may also be a carbohydrate moiety similar to galactomannan or galactofuran.

We manufactured an immunochromatography test strip using this monoclonal antibody 0014. We obtained good results when we examined the ability of this test to detect dermatophytes in 37 nail specimens from patients with tinea unguium and healthy volunteers. Thus, the KOH direct microscopy method revealed fungal hyphae in the sample of the antibody reactive positive case (case no. 30) from the healthy volunteer group (data not shown); this case could therefore be considered as a suspected potential patient. One case (case no. 3) of the patient group was negative. The cause of this negative antibody reactivity might be nail candidiasis. It is very difficult to distinguish nail candida and tinea unguium by the KOH direct microscopy method. The culture method can provide such distinction, but the culture method was not performed in the present study.

Limitations of this report are that the number of cases examined in this study is small and that the cases are limited to our hospital. It will therefore be necessary to examine this test strip using many more cases as a multicentre trial in the future. Moreover, in the present study, we used nail samples from healthy volunteers as negative controls. However, it will be necessary to use the test strip to examine deformed and discoloured nails that are difficult to distinguish from onychomycosis.

Regarding onychomycosis, the guidelines of the UK state that laboratory confirmation of a clinical diagnosis of tinea unguium should be obtained before starting treatment for several reasons.^19^ The standard for diagnosis of onychomycosis is a positive result on microscopical examination and in culture of nail clippings. However, the culture method has poor sensitivity, with false negative rates of 30 to 50%.^20^ The positive detection rate as assessed using the KOH direct microscopy method is high; however, not all dermatologists have the skill required for sampling from the nail, processing the sample, and recognising fungal elements in the specimen. These factors mean that a new detection method is required. The diagnosis of tinea unguium by using the newly manufactured immune-chromatographic test strip of the present study is expected to have both high sensitivity and specificity, and therefore clinical application of the immune-chromatographic test strip to diagnosis of tinea unguium is expected in the future.

In the immune-chromatographic test strip examination of nail from patients with Trichophytosis unguium, strong reactive bands were found in the majority of cases, although some cases (case nos.13, 14, 15, and 17) displayed weakly reactive bands. Examination of the sensitivity of the immune-chromatographic test strip indicated that the strength of the band reflected the antigen level. Therefore, semi-quantitative analysis of the fungus may be enabled by measurement of the density of the band using densitometry.

In this study, we use heat treatment for extraction of the soluble antigen from the clinical specimen. However, Noriki has succeeded in extraction of the antigen by non-ionic detergents without heat-treatment.^16,17^ Non-heat treatment extraction of the antigen allows extraction without using a heating tool and reduces the risk of burn, which would make this diagnostic tool easy to use.

In the present study, we only showed examination of specimens of nail from patients with tinea unguium. However, examination of dermatophytes using the scale from patients with tinea pedis has also been reported. Higashi et al. reported that the sensitivity and the specificity of a test strip was 82.1% and 76.2%, respectively, for 88 cases of tinea pedis in a comparison between KOH direct microscopy methods and an immune-chromatographic test strip method.^21^ Tsunemi et al. has reported regarding screening for tinea unguium and tinea pedis using an immune-chromatographic test strip method.^22,23^ Examination using a larger number of samples will be required for examination of tinea pedis.

Additionally, the monoclonal antibody of our study also reacts with *M. canis* which causes most of the tinea of cats and dogs. It is therefore possible that this test strip could be applied to diagnosis of the tinea of cats and dogs.
