# Supplementary material for: Production of an anti-dermatophyte monoclonal antibody and its application: immunochromatographic detection of dermatophytes
Source: Med Mycol. 2016 Jun 1;54(8):808–15. doi: 10.1093/mmy/myw037 (PMC5057457; doi:10.1093/mmy/myw037)
Supplement: SUPPLEMENTARY MATERIAL [file supp_54_8_808__index.html]

Production of an anti-dermatophyte monoclonal antibody and its application: immunochromatographic detection of dermatophytes — Production of an anti-dermatophyte monoclonal antibody and its application: immunochromatographic detection of dermatophytes — SUPPLEMENTARY MATERIAL 

# Production of an anti-dermatophyte monoclonal antibody and its application: immunochromatographic detection of dermatophytes

## SUPPLEMENTARY MATERIAL

- SUPPLEMENTARY MATERIAL
