# Supplementary figures and images for: Production of an anti-dermatophyte monoclonal antibody and its application: immunochromatographic detection of dermatophytes
Source: Med Mycol. 2016 Jun 1;54(8):808–15. doi: 10.1093/mmy/myw037 (PMC5057457; doi:10.1093/mmy/myw037)

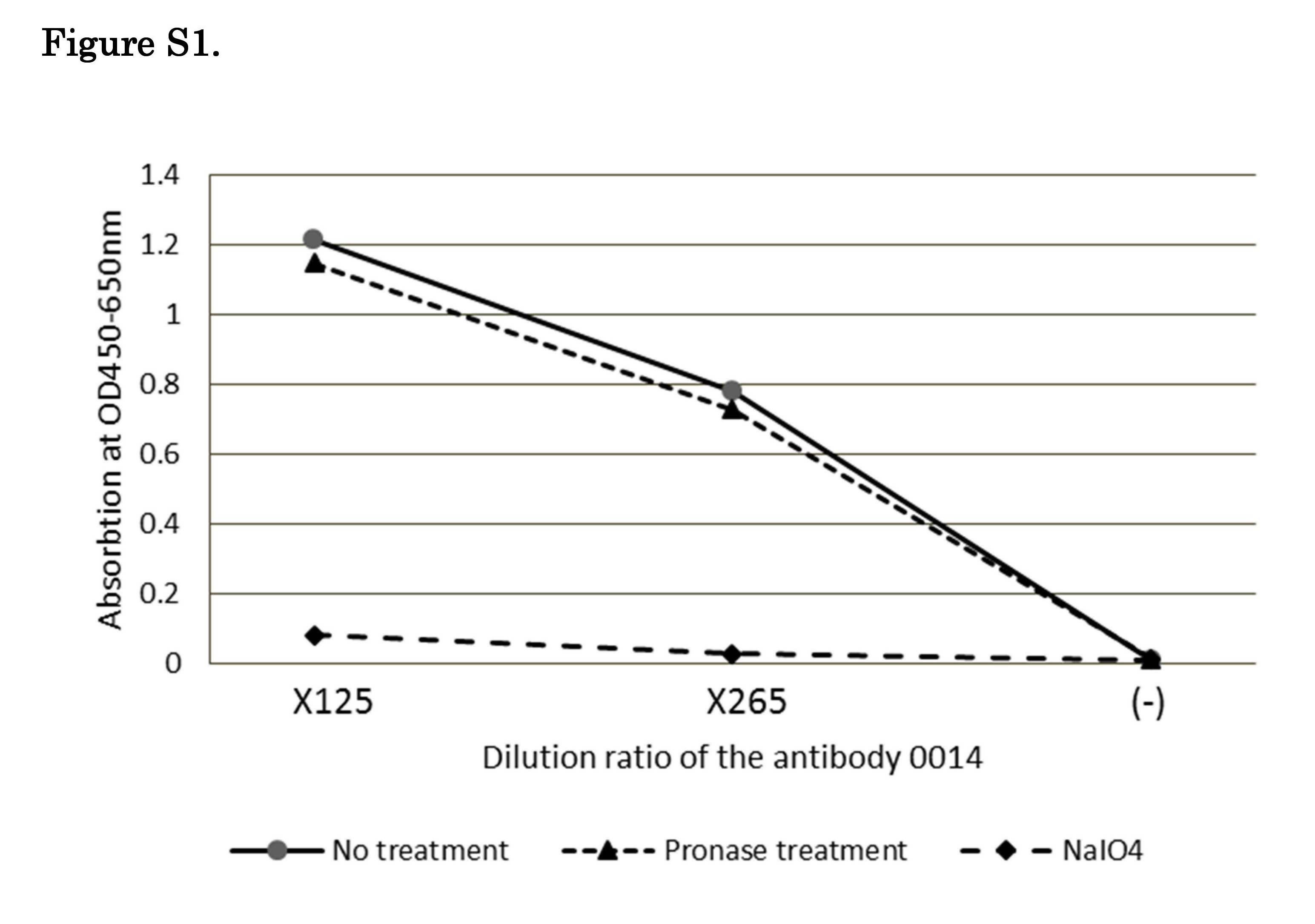

Supplement: SUPPLEMENTARY MATERIAL [file supp_myw037_mm-2016-0035-File005.jpg]
